# An evaluation of the stability of image quality parameters of Elekta X‐ray volume imager and iViewGT imaging systems

**DOI:** 10.1002/acm2.12289

**Published:** 2018-03-09

**Authors:** Dennis N. Stanley, Karl Rasmussen, Neil Kirby, Nikos Papanikolaou, Alonso N. Gutiérrez

**Affiliations:** ^1^ University of Texas Health Science Center San Antonio San Antonio Texas USA; ^2^ Miami Cancer Institute, Baptist Hospital Miami FL USA

**Keywords:** Elekta iView, Elekta XVI, image stability, image Quality, temporal trending

## Abstract

**Introduction:**

A robust image quality assurance and analysis methodology for image‐guided localization systems is crucial to ensure the accurate localization and visualization of target tumors. In this study, the long‐term stability of selected image parameters was assessed and evaluated for the cone‐beam computed tomography (CBCT) mode, planar radiographic kV mode, and the radiographic MV mode of an Elekta VersaHD.

**Materials and Methods:**

The CATPHAN, QckV‐1, and QC‐3 phantoms were used to evaluate the image quality parameters. The planar radiographic images were analyzed in PIPSpro™ with spatial resolution (f30, f40, f50), contrast to noise ratio (CNR) and noise being recorded. For XVI CBCT, Head and Neck Small20 (S20) and Pelvis Medium20 (M20) standard acquisition modes were evaluated for uniformity, noise, spatial resolution, and HU constancy. Dose and kVp for the XVI were recorded using the Unfors RaySafe Xi system with the R/F low detector for the kV planar radiographic mode. For each metric, values were normalized to the mean and the standard deviations were recorded.

**Results:**

A total of 30 measurements were performed on a single Elekta VersaHD linear accelerator over an 18‐month period without significant adjustment or recalibration to the XVI or iViewGT systems during the evaluated time frame. For the planar radiographic spatial resolution, the normalized standard deviation values of the f30, f40, and f50 were 0.004, 0.003, and 0.003 and 0.015, 0.009, and 0.017 for kV and MV, respectively. The average recorded dose for kV was 67.96 μGy. The standard deviations of the evaluated metrics for the S20 acquisition were 0.083(f30), 0.058(f40), 0.056(f50), 0.021(Water/poly‐HU constancy), 0.029(uniformity) and 0.028(noise). The standard deviations for the M20 acquisition were 0.093(f30), 0.043(f40), 0.037(f50), 0.016(Water/poly‐HU constancy), 0.010(uniformity) and 0.011(Noise).

**Conclusion:**

A study was performed to assess the stability of the basic image quality parameters recommended by TG‐142 for the Elekta XVI and iViewGT imaging systems. The two systems show consistent imaging and dosimetric properties over the evaluated time frame.

## 1 INTRODUCTION

As image‐guided radiation therapy (IGRT) systems become the clinical standard of care for many treatment sites, a need for a high standard of image quality assurance is essential to ensure better localization and identification of regions of interest, particularly tumor volumes. IGRT, when compared to non‐image‐guided techniques, offers an enhanced delivery accuracy of volumetric dose distributions,^1^ enables intra‐ and interfraction visualization, identification of the target volume^2^ and the potential to reduce patient specific PTV (planning target volume) margins.^3^, ^4^, ^5^

The American Association of Physicist in Medicine (AAPM) Task Groups 142^5^ and 179^6^ have discussed the capabilities and set basic image quality QA procedures for both planar radiographic and cone‐beam computed tomography (CBCT) imaging modalities. Task Group 142 recommends a QA testing program, frequency and tolerance values for the planar radiographic modalities,^5^ while TG 179 recommends a similar format for all CBCT‐based imaging modalities.^6^ In both reports, the necessity for a clinically robust QA program that maximizes image quality while minimizing radiation dose is imperative to ensure functionality and the consistency of IGRT equipment. Both reports stipulate only that a tolerance of “baseline” is needed, however, neither task group reports proposed a protocol for defining this “baseline.” This demonstrates a need for an institutionally specific initial setup and monitoring program for QA and safety. A recent publication^7^ detailed the stability of the Varian IGRT systems, but an analysis and comparison with the Elekta IGRT systems was not available at that time. With this in mind, the aim of this study was to evaluate the stability of the image quality parameters of the Elekta X‐ray volume imager (XVI) and iViewGT imaging systems using methods previously published.^7^ Using these methods, an analysis of the consistency and stability over the evaluated time period can be performed. Using this information, institutional QA tolerances for warning and action thresholds for each imaging quality parameter can be established and compared against the reported image quality metrics of the Varian OBI.

## 2 MATERIALS/METHODS

### 2.A Materials

#### 2.A.1 Elekta X‐ray volume imaging system

**Figure 1:**
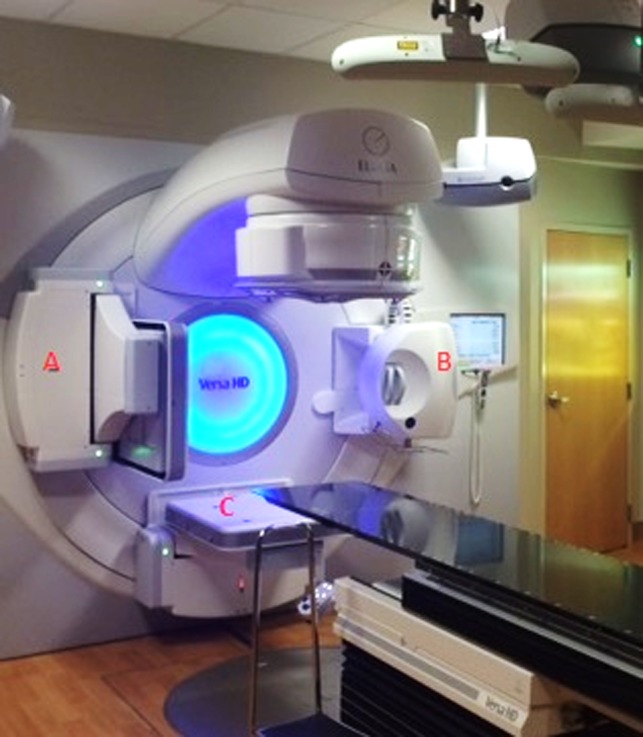
The X‐ray volume imaging (XVI) guidance system and iViewGT image system of the Elekta VersaHD radiation delivery system are shown. (a) a‐Si flat panel detector of the XVI, (b) kV X‐ray source of the XVI and (c) iViewGT imaging panel.

The Elekta XVI system (Elekta, Crawley, UK) consists of two gantry mounted robotic arms that are mounted perpendicular to the radiation beam designated at (a) and (b) in Fig. 1. The detector for the XVI is a‐Si flat panel detector with an active imaging area of 42.5 × 42.5 cm. The XVI is capable of utilizing a small, medium, or large fields of view (FOV) for different anatomical sites. Commonly, a small FOV will be used with a 200° rotation (comparable to a full‐fan CBCT) for imaging of the head or neck while the medium FOV (Half‐fan CBCT) is standardly used for larger sites. When a medium or large FOV is selected, the detector panel is shifted 11.5 cm and 19 cm, respectively, from the central axis of the kV X‐ray beam (the small FOV is obtained by centering the detector pane).^8^ The XVI contains preset parameters that are configured per anatomical site for imaging geometry, beam characteristics, and reconstruction method. It also allows for customization of the tube potential, number of frames, mA and ms per frame, start and stop gantry angles, and reconstruction resolution (1‐mm pixel size for medium resolution and 0.5‐mm pixel size for high resolution). For this portion of the study, a 200° gantry rotation with small FOV will be analyzed along with a 360° gantry rotation with a medium FOV.

#### 2.A.2 Elekta iViewGT electronic portal imaging device

The Elekta iViewGT (Elekta, Crawley, UK) is an amorphous silicon flat panel imaging device mounted on a robotic arm designated at C in Fig. 1. This arm allows the detector to be positioned at source to electronic portal imaging device (EPID) distance of 160 cm with an active imaging area of 41 × 41 cm.^9^ The image matrix is created from an array of 1024 × 1024 photodiodes with a pitch of 400 μm.^10^ While the EPID can be operated in various acquisition modes, a single exposure, 6 MV planar radiographic mode was used in this study.

#### 2.A.3 The CATPHAN^®^ 504 Phantom

The CATPHAN 504 (Phantom Laboratory, Salem, NY, USA) was used to evaluate the image quality parameters of the kV‐CBCT for both small and medium acquisition modes. The CATPHAN is a cylindrical phantom with outer diameter of 20 cm, inner diameter of 15 cm and 4 different inserted modules that can evaluate image uniformity, image noise, image high contrast spatial resolution, HU constancy, geometric distortion, and slice thickness.^11^ The CATPHAN was chosen for its ease of setup and use, commercial availability (commonly provided with purchase of linear accelerator) and compatibility with the PIPSpro software.

#### 2.A.4 QCkV‐1, QC‐3 Phantoms, and PIPSpro^™^ V 5.2‐5.3

The PIPSpro QA software and phantom package (Standard Imaging, Middleton, WI, USA) was used in this study to analyze the specific image quality parameters for both the XVI and iViewGT. PIPSpro was chosen because it has a dedicated kV X‐ray phantom (QCkV‐1 Phantom), dedicated MV X‐ray phantom (QC‐3), software tracking capabilities and its widespread use for TG‐142 imaging analysis. For the kV and MV planar radiographic modes, TG‐142 imaging metrics can be measured and analyzed in PIPSpro using the QCkV‐1 and QC‐3 phantoms: high contrast spatial resolution, contrast to noise ratio (CNR), and image noise. For the CBCT, the TG‐179 imaging parameters can also be measured and analyzed in PIPSpro with the CATPHAN phantom: image uniformity, image noise, high contrast spatial resolution, HU constancy, image geometric distortion, and slice thickness. The QCkV‐1 and QC‐3 phantoms have 11 different regions of interest that contain line pair patterns and materials of varying densities.^3^ Having these different regions of the phantoms allow the PIPSpro software to evaluate, store and track the image quality parameters over time. The current version (Version 5.3) of PIPSpro software offers two analysis options: (1) acquire a flood field and an image of the QCkV‐1 or QC‐3 phantoms or (2) acquire two sequential phantom images. In this study, the images were evaluated using an acquired flood field and one image of the phantom.

#### 2.A.5 Unfors RaySafe Xi R/F and CT Detectors

The Unfors RaySafe Xi (Unfors RaySafe AB, Billdal, Sweden) is a comprehensive system of detectors that can perform multi‐parameter measurements on all X‐ray modalities. The system is composed of a base unit and multiple detectors that are jointly certified by the AALA (American Association for Laboratory Accreditation) and ADCL (American dosimetry calibration laboratory). In this study, the R/F was used in conjunction with the base unit for kV planar radiographic mode. The R/F detector is a small, lightweight, portable, and wireless detector capable of measuring kVp, dose, dose rate, pulse, pulse rate, dose/frame, time, half value layer, total filtration and waveforms simultaneously. For the purposes of this study, the image parameters evaluated were the dose and the X‐ray energy for the kV planar radiographic mode.

### 2.B Methods

#### 2.B.1 kV planar radiographic

**Figure 2:**
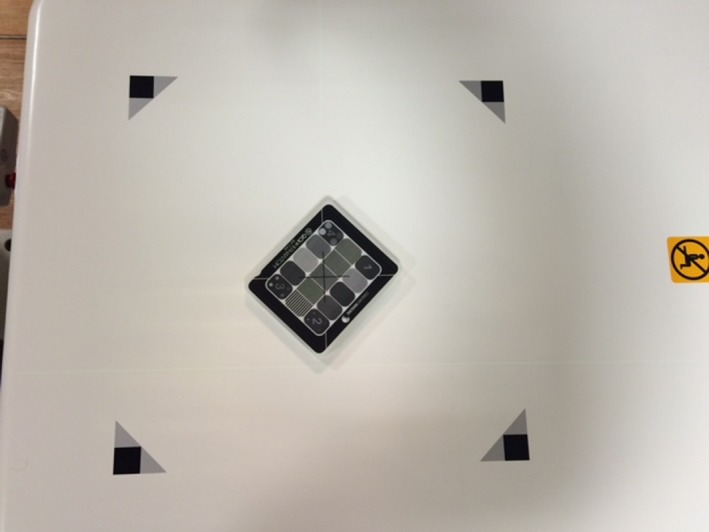
The a‐Si flat panel detector of the Elekta XVI system is shown with the (a) QCkV‐1 image quality phantom.

To evaluate the imaging quality parameters, the QCkV‐1 phantom was placed directly onto the face of the XVI detector with the F0/S20 inserts and aligned to the room lasers as seen in Fig. 2. One image was acquired with the following settings: 70 kV, 160 mA, and 200 ms. After removing the QCkV‐1 phantom, a second flood field image was acquired with the same settings as before. The two images were then analyzed in PIPSpro™ and the high contrast spatial resolution, noise and contrast to noise ratio were recorded. Each image has three separate values of the high contrast spatial resolution (f30, f40, f50(lp/mm)), which represent the frequencies at 30%, 40% and 50% of the maximum for the relative modulation transfer function (RMTF). Next, the Unfors RaySafe Xi R/F detector was placed onto the XVI detector. The process was repeated with the dose and X‐ray energy being manually recorded after each acquisition.

#### 2.B.2 MV planar radiographic

**Figure 3:**
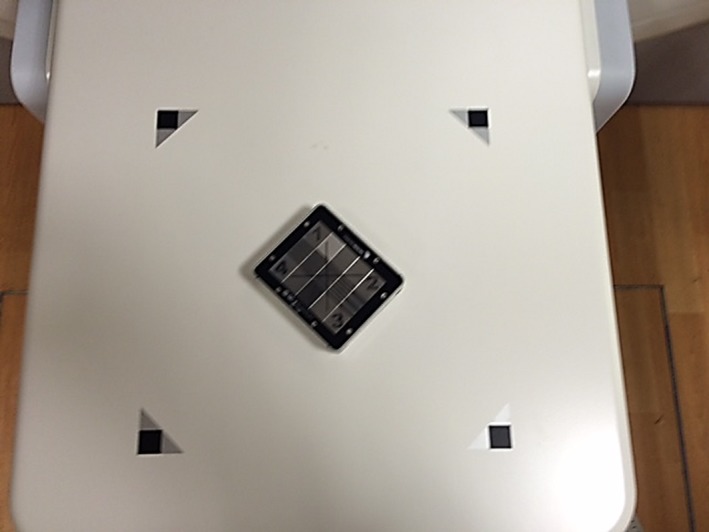
The iVieiwGT flat panel detector is shown with the (a) QC‐3 image quality phantom in measurement position.

To evaluate the imaging quality parameters, the QC‐3 phantom was placed directly onto the face of the iViewGT EPID and aligned to the room lasers as seen in Fig. 3. The first image was acquired at 6 MV with 4 MU and a 14 × 14 cm field size. After removing the QC‐3 phantom, a second flood field image was acquired with 4 MU and an open field that covered the total active imaging area of the iViewGT EPID. The two images were then analyzed in PIPSpro™ with the high contrast spatial resolution, noise and contrast to noise ratio being recorded.

#### 2.B.3 kV‐CBCT

For the kV‐CBCT image quality parameters, the CATPHAN was cantilevered over the edge of the couch according to manufactures specifications. The CATPHAN was leveled and positioned to the imaging isocenter with the aid of the in room localization lasers. One kV‐CBCT scan per image setting was acquired with the specific settings listed in Table 1. The image volumes were exported via DICOM protocol and then were analyzed in PIPSpro with specific image quality parameters being evaluated. For statistical analysis, the QI Macros (KnowWare International Denver, CO, USA) add‐on statistical analysis package (v2010.11) was used in Microsoft Excel. The variable control charts module was used to analyze the quality control processes using an X‐bar chart (individual moving range chart test). The software provides control limits for the data and establishes which data points are in and out of control processes.

**Table 1:** Image quality scanning parameters for the Elekta VersaHD XVI kV‐CBCT

	Small	Medium
CBCT mode	Head and Neck S20	Pelvis M20
Start angle	25	180
Stop angle	180	180
Reconstructed volume	512 × 512	512 × 512
kV collimator	S20	M20
kV filter	F0	F1
kV	100	120
mA per frame	10	40
ms per frame	10	40
Frames	183	660

## 3 RESULTS

**Figure 4:**
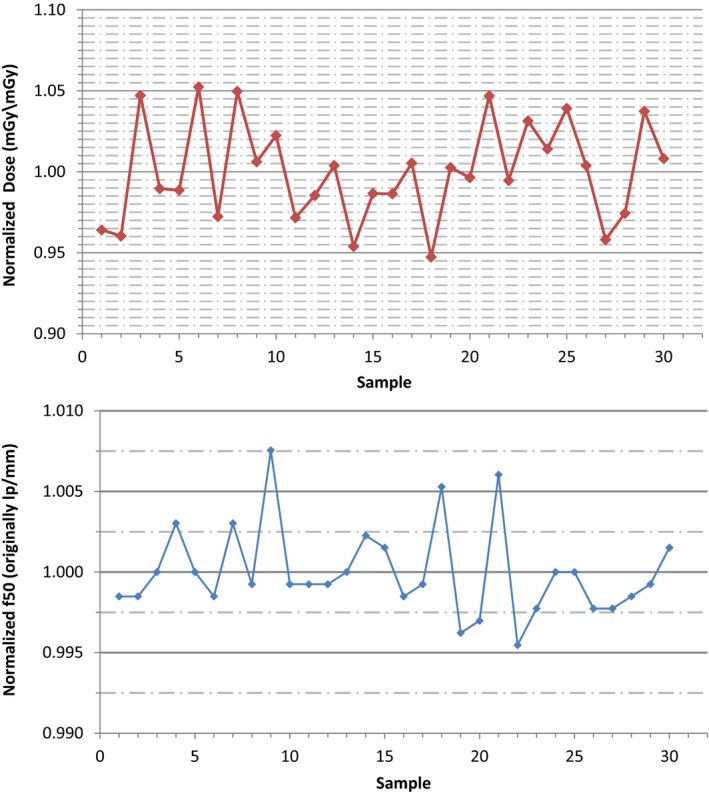
(top) The run chart of the normalized f_50_ values measured with the QCkV‐1 phantom and PIPSpro software is shown for the kV planar radiographic mode of the Elekta XVI. (bottom) The run chart of the normalized image dose values measured with the Unfors RaySafe Xi R/F detector is shown for the kV planar radiographic mode of the Elekta XVI.

A total of 30 measurements were performed on a single Elekta VersaHD linear accelerator over an 18‐month period without significant adjustment or recalibration to the XVI or iViewGT systems during the evaluated time frame. For each image quality parameter, measured values were normalized to the mean and the standard deviations were recorded. Table 2 shows the standard deviations of all the image quality parameters evaluated for the kV planar radiographic, MV planar radiographic, and kV‐CBCT modes. Run charts were created for each of the evaluated parameters to characterize the temporal variability of each parameter over the evaluated time period and establish upper and lower control limits. Figure 4 shows a sample run chart for the normalized f50 and normalized dose values of the planar kV planar radiographic mode. In general, all of the data for the other evaluated parameters showed similar temporal trending to that in Fig. 4.

**Table 2:** Normalized standard deviations for all evaluated metrics

Planar radiographic							
kV				MV			
f30(lp/mm)	0.004	Noise	0.048	f30(lp/mm)	0.015	Noise	0.005
f40(lp/mm)	0.003	CNR	0.024	f40(lp/mm)	0.009	CNR	0.021
f50(lp/mm)	0.003			f50(lp/mm)	0.017		
kVp	0.006	Dose(μGy)	0.030				
CBCT							
Small							
High contrast spatial resolution (lp/mm)		HU constancy		Noise		Uniformity	
f30	0.083	Lung(PMP)	0.049	Mean	0.029	Mean	0.028
f40	0.058	Water(Poly)	0.021	Sigma	0.059	Sigma	0.053
f50	0.056	Air	0.025				
Medium							
High contrast spatial resolution (lp/mm)		HU constancy		Noise		Uniformity	
f30	0.093	Lung(PMP)	0.010	Mean	0.010	Mean	0.011
f40	0.043	Water(Poly)	0.016	Sigma	0.041	Sigma	0.032
f50	0.037	Air	0.006				

Following the precedent set by Stanley et al^7^ tolerance thresholds were based on 1σ and 2σ standards. The warning threshold is chosen to alert the user of a potential abnormal deviation of that image quality parameter. A single measurement deviation should not require an action to be taken, but should serve as an alert for closer monitoring. If the image quality parameter value exceeds the 2σ threshold, the parameter value is significantly different from the intrinsic variation of the temporal data and should serve as an action threshold. The action to be taken is dependent upon the underlying cause of the deviation and the clinical impact of the deviation. Table 3 shows the warning and action tolerances adopted in our institution for the kV/MV planar radiographic modes. Tables 4 and 5 show the warning and action tolerances adopted in our institution for the small and medium kV‐CBCT modes, respectively.

**Table 3:** Image quality consistency thresholds for the planar radiographic modalities

kV			MV		
	Warning (%)	Action (%)		Warning (%)	Action (%)
f30(lp/mm)	1	3	f30(lp/mm)	2	4
f40(lp/mm)	1	3	f40(lp/mm)	1	3
f50(lp/mm)	1	3	f50(lp/mm)	2	4
Noise	5	10	Noise	1	2
CNR	3	6	CNR	2	4
Dose	3	6			
kVp	1	2			

Sample size of 30 measurements.

**Table 4:** Image quality consistency thresholds for the small(S20) CBCT

	Warning (%)	Action (%)
High contrast spatial resolution(lp/mm)		
f30	8	16
f40	5	11
f50	5	11
HU constancy		
Lung(PMP)	5	9
Water(Poly)	3	6
Air	2	5
Uniformity		
Mean	3	6
Sigma	5	10
Noise		
Mean	3	6
Sigma	6	11

Sample size of 30 measurements.

**Table 5:** Image quality consistency thresholds for the Medium (M20) CBCT

	Warning (%)	Action (%)
High contrast spatial resolution(lp/mm)		
f30	9	18
f40	4	9
f50	4	8
HU constancy		
Lung(PMP)	2	4
Water(Poly)	2	4
Air	1	3
Uniformity		
Mean	1	3
Sigma	3	6
Noise		
Mean	1	3
Sigma	4	8

Sample size of 30 measurements.

## 4 DISCUSSION

With the growth of the modern state‐of‐the‐art image guidance systems for use in IGRT, the evaluation of accurate temporal stability has become important to ensuring overall imaging consistency. AAPM TG‐142 and TG‐179 address the consistency of systems by recommending set of annual, monthly and daily QA assessments of specific image quality parameters. Although the AAPM task group reports, along with the IGRT Medical Physics Practice Guidelines (MPPGs),^12^ convey a comprehensive review of the image quality QA for an IGRT system they fail to appropriately define the methodology of the establishment of the “baseline” and recommended tolerance levels. A recent publication^7^ presented a thoughtful and comprehensive analysis of the reasoning behind and importance of the tolerance threshold delineation of 1σ and 2σ. Based on the established methodology of this publication, a similar analysis of the imaging systems of a comparable linear accelerator, The VersaHD, was performed to evaluate whether a difference in temporal stability existed between the two imaging systems. Tables 6, 7, 8 show an analysis of comparable image quality metrics between the Elekta XVI and Varian OBI for the planar radiographic modalities, small/Full‐Fan kV‐CBCT, and Medium/Half‐fan kV‐CBCT, respectively. It should be noted that this comparison was not done to establish a preference for one system but to report our tolerance values of key image quality parameters for our XVI and iViewGT systems of the VersaHD with respect to the information already published on the Varian OBI and EPID imaging systems of the Novalis Tx. These tolerance values “are strictly dependent on the observed behavior of the image quality parameter rather than on a threshold derived based on a specific clinical impact” as there is little published evidence to the later.^7^ Although the observed behavior of the image quality parameters will be institution and machine specific, and should be quantified by each individual institution, an analysis of the methodology was done with a partnering facility. This facility has one Elekta VersaHD and used the same types of image quality phantoms and analysis. Initial tolerance levels were established using the results and methodology of this study and based on the temporal trending, these initial tolerance levels are still appropriate.

**Table 6:** Comparison of Image quality action thresholds for the planar radiographic modalities between the XVI and OBI

	XVI (%)	OBI* (%)
kV		
High contrast spatial resolution(lp/mm)		
f30	3	4
f40	3	3
f50	3	3
Dose	6	2
kVp	2	2
MV		
High contrast spatial resolution(lp/mm)		
f30	4	4
f40	3	3
f50	4	3

*Data from Stanley et al.^7^

**Table 7:** Comparison of image quality action thresholds for the Small (S20) and Full‐Fan CBCT

	XVI (%)	OBI* (%)
High contrast spatial resolution(lp/mm)		
f30	16	18
f40	11	18
f50	11	16
HU constancy		
Lung(PMP)	9	6
Water(Poly)	6	12
Uniformity	10	12
Noise	11	12

*Data from Stanley et al.^7^

**Table 8:** Comparison of image quality action thresholds for the Medium (M20) and Half‐Fan CBCT

	XVI (%)	OBI* (%)
High contrast spatial resolution(lp/mm)		
f30	18	20
f40	9	22
f50	8	34
HU constancy		
Lung(PMP)	4	8
Water(Poly)	4	12
Uniformity	6	17
Noise	8	16

*Data from Stanley et al.^7^

Ultimately, the clinical impact of these deviations will be of significance when these imaging systems become utilized more for adaptive radiotherapy. The effect of the temporal variance of the image quality metrics of the kV‐CBCT could play a role in dose reconstruction and delineation of target volumes in adaptive radiotherapy, based on restrictive constraints on the image quality. To date, a few publications^8^, ^13^, ^14^, ^15^, ^16^ have analyzed the effect of various image quality metrics in CT and CBCT but these evaluated differences are much larger in scale to the temporal deviations evaluated in this study. More investigation into the effect of these temporal differences and threshold limits is needed to quantify the effect an out of tolerance measurement would have clinically. As the image quality and technology of CBCT continues to improve, the clinical impact of the temporal image quality deviations needs further evaluation. In general, technological advances, including advances in detector design, generator output consistency or image reconstruction algorithm, will require careful consideration on a case by case basis as to the effect on the clinically established baselines.

## 5 CONCLUSION

A study of the stability for image quality parameters of Elekta XVI and iViewGT imaging systems was performed using commercially available imaging QA phantoms and software with a total of 30 measurements over an 18‐month period. Run charts were created for each of the evaluated parameters. Both systems, for each image quality parameter, show consistent imaging and dosimetric properties over the evaluated time frame for the normalized mean and standard deviations, as well as comparable results to previously completed studies.^7^, ^8^, ^9^

## CONFLICT OF INTEREST

The authors have no conflict of interest to report.
